# Predictive Performance Models in Long-Distance Runners: A Narrative Review

**DOI:** 10.3390/ijerph17218289

**Published:** 2020-11-09

**Authors:** José Ramón Alvero-Cruz, Elvis A. Carnero, Manuel Avelino Giráldez García, Fernando Alacid, Lorena Correas-Gómez, Thomas Rosemann, Pantelis T. Nikolaidis, Beat Knechtle

**Affiliations:** 1 Faculty of Medicine, University of Málaga, Andalucía TECH, 29071 Málaga, Spain; alvero@uma.es; 2 Translational Research Institute for Metabolism and Diabetes, Florida Hospital Sanford, Orlando, FL 32804, USA; Elvis.AlvarezCarnero@adventhealth.com; 3 Sanford Burnham Prebys Medical Discovery Institute, La Jolla, CA 92037, USA; 4 Faculty of Sports Science and Physical Education, University of A Coruña, 15179 Oleiros, Spain; manuel.avelino.giraldez.garcia@udc.es; 5 Department of Education, Health Research Centre, University of Almería, 04120 Almería, Spain; falacid@ual.es; 6 Faculty of Education Sciences, University of Málaga, Andalucía TECH, 29071 Málaga, Spain; lcg@uma.es; 7 Institute of Primary Care, University of Zurich, 8006 Zurich, Switzerland; thomas.rosemann@usz.ch (T.R.); beat.knechtle@hispeed.ch (B.K.); 8 School of Health and Caring Sciences, University of West Attica, 12243 Athens, Greece

**Keywords:** prediction equations, performance, long-distance runners

## Abstract

Physiological variables such as maximal oxygen uptake (VO_2_max), velocity at maximal oxygen uptake (*v*VO_2_max), running economy (RE) and changes in lactate levels are considered the main factors determining performance in long-distance races. The aim of this review was to present the mathematical models available in the literature to estimate performance in the 5000 m, 10,000 m, half-marathon and marathon events. Eighty-eight articles were identified, selections were made based on the inclusion criteria and the full text of the articles were obtained. The articles were reviewed and categorized according to demographic, anthropometric, exercise physiology and field test variables were also included by athletic specialty. A total of 58 studies were included, from 1983 to the present, distributed in the following categories: 12 in the 5000 m, 13 in the 10,000 m, 12 in the half-marathon and 21 in the marathon. A total of 136 independent variables associated with performance in long-distance races were considered, 43.4% of which pertained to variables derived from the evaluation of aerobic metabolism, 26.5% to variables associated with training load and 20.6% to anthropometric variables, body composition and somatotype components. The most closely associated variables in the prediction models for the half and full marathon specialties were the variables obtained from the laboratory tests (VO_2_max, *v*VO_2_max), training variables (training pace, training load) and anthropometric variables (fat mass, skinfolds). A large gap exists in predicting time in long-distance races, based on field tests. Physiological effort assessments are almost exclusive to shorter specialties (5000 m and 10,000 m). The predictor variables of the half-marathon are mainly anthropometric, but with moderate coefficients of determination. The variables of note in the marathon category are fundamentally those associated with training and those derived from physiological evaluation and anthropometric parameters.

## 1. Introduction

The great popularity of long-distance running has seen an unprecedented increase in the last 10 years. This has generated, in coaches and athletes, a great interest in the development of performance prediction models based on linear regression equations, with the aim of helping many athletes in their preparation for competitions. These predictions are based on a combination of physiological, anthropometric, nutritional and training factors (modifying frequency, volume and intensity), most obtained in exercise physiology laboratories, through variables related to training load [1,2].

Performance in long-distance disciplines can be defined as the final time or race time, and its understanding is important both for designing training programs and for determining scheduled training and race pace. However, accurate knowledge is frequently difficult to obtain, especially in long-distance races, as it would involve high training loads, which can, at times, indicate poor race planning in inexperienced runners who normally use polarized training methods [3]. This and other factors associated with the control of training, result in predictive models being recognized and useful for coaches or professional runners. The physiological adaptations produced by training in amateur runners are well understood and are generally those performed at submaximal intensities with continuous training strategies [4]. In high-level athletes, these improvements are seen particularly with tempo runs and short-interval training, as methods to improve performance [5]. Therefore, transferring the results and conclusions obtained from amateur athletes to high-level athletes is not advisable [6].

Performance in endurance running is influenced by a variety of factors, both anthropometric and training. Morphological (somatotype components) and anthropometric characteristics such as skinfolds, body fat percentage, circumferences, lower limb length, weight, height and body mass index appear to influence performance. Accordingly, certain characteristics have a better relationship between energy expenditure and performance [7,8].

There are numerous studies on physiological factors in the literature on performance prediction in long-distance runners. Classically, maximal oxygen uptake (VO_2_max), running economy (RE) and anaerobic threshold (AT) stand out as the main variables that have been used to predict performance in long-distance races [9,10], but a large gap exists in the field of performance prediction based on field tests.

The aim of this narrative review was to undertake a descriptive, analytical and detailed analysis of the determinants and predictive ability of anthropometric, physiological (laboratory test), training and combined variables, as well as field assessments (field tests), to estimate performance in specialties of long-distance races (5000 m, 10,000 m, half–marathon and marathon).

## 2. Materials and Methods

This document is classified as a narrative review and was carried out under a framework of assigning key attributes based on Search, Appraisal, Synthesis and Analysis (SALSA) [11]. Accordingly, the search was exhaustive. The synthesis is a tabular exposition of the data and the analysis may be chronological, conceptual or thematic [11]. In general terms, this narrative review presents all the known published works that include runners of different levels: all of these in different types of runner (amateur, moderately trained, highly trained, high-level and elite) with the common denominator that they are generally trained both in length of time and number of weekly sessions. Also included are all studies that found associations between anthropometric and physiological parameters and performance in the middle-distance (5000 m and 10,000 m) and long-distance (half-marathon and marathon) events.

### 2.1. Search

The abstracts of original English articles registered in the Pubmed, SciELO (Scientific Electronic Library On line), ScienceDirect and SportDiscus databases were reviewed. The terms entered in the search engines were as follows: “runners”, “long distance runners”, “performance”, “performance prediction”, “anthropometric”, “physiological determinants”, “performance determinants”, “5000 m”, “10,000 m”, “half-marathon” and “marathon”, as well as the combinations of all of them, depending on the specialty examined.

### 2.2. Selection Criteria

The selection criteria were all relevant articles, as well as books and monographs. The first evaluation consisted of reading the abstract and the full text of the selected studies, followed by an analysis of the results.

### 2.3. Exclusion Criteria

Case studies, duplicate articles and abstracts without clear and sufficient information were excluded.

## 3. Results

**Figure 1:**
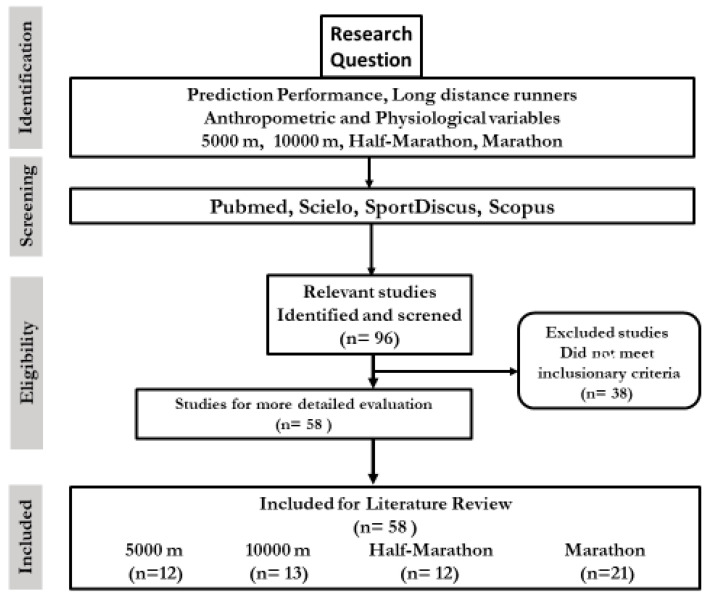
Diagram of study search and selection process.

The flow chart (Figure 1) shows the final selection of 58 articles, with 12 articles identified for the 5000 m modality, 13 for the 10,000 m, 12 for the half-marathon and 21 for the marathon.

In Table 1, the variables are grouped as demographic, laboratory assessments, field test, training, anthropometric and others.

### 3.1. Demographic Variables

Of the seven demographic variables, the most notable is age, which is included in all the specialties studied. Gender is only recorded in the 5000 m specialty [12].

### 3.2. Aerobic Metabolism Assessment Variables

In this section, the variables were classified into two groups:

1. Maximum range (VO_2_max, velocity at maximal oxygen uptake [*v*VO_2_max], maximum heart rate, maximum lactate, *v*VO_2_ with the University of Montreal Track Test, anaerobic capacity and oxygen deficit, etc.).

2. Submaximal range (VO_2_ at lactate threshold, lactate threshold, velocity at lactate levels of 2.5–3 and 4 mmol/L, RE, heart rate at individual anaerobic threshold (IAT), velocity at heart rate deflection point, VO_2_ and % VO_2_ at AT, velocity at AT, lactate level at AT and % of peak velocity at AT). Of particular note are *v*VO_2_max and VO_2_max, RE, understood as oxygen uptake at specific velocity, VO_2_ at AT and velocity at the level of 4 mmol/L lactate. Thirty-one of these studies include mL/kg/min among the variables that are associated with or are predictive factors of running performance from middle to long distance. Additionally, 24 studies include variables such as km/h, m/min, m/s associated with conditions obtained at VT2 (anaerobic threshold), velocity at heart rate deflection, IAT, ATLab (AT in laboratory test), etc.

### 3.3. Training Variables

The training variables were grouped into two categories: quantitative (mean race duration, number of training sessions per week, miles per week, km per week, training volume, miles in 8 weeks, training in 9 weeks, years of training) and qualitative (training pace, record for 1 mile, 5 miles, 10 miles, half-marathon time and having finished a marathon).

### 3.4. Field Test Variables

Only two studies measuring AT using the University of Montreal Track test [13], and covered distance in the Cooper test [14,15]

### 3.5. Anthropometric Variables

These variables are classified into three categories: (i) basic measurements (height, weight, body mass index, skinfolds and muscle circumferences), (ii) body composition fractions (fat mass, fat-free mass and skeletal muscle mass) and (iii) somatotype components (endomorphy, mesomorphy and ectomorphy). Other important performance-related variables are body mass index, fat mass percentage, and skinfolds as regional indicators of adiposity associated with performance. Fifteen of the 26 studies were conducted in the half-marathon specialty by Knechtle’s research group [8,16,17].

### 3.6. Other Variables

Noteworthy are also the use of a biochemical variable such as transferrin levels, as well as a model based on data collection through a post-competition survey [14] and leg volume and heart rate changes during the Ruffier test recovery period [15].

### 3.7. Data Management and Presentation

Table 2, Table 3, Table 4 and Table 5 are individual tables for each distance (5000 m, 10,000 m) and long-distance specialty (half-marathon and marathon) respectively and structured to display: Author, year of publication, sex, number of participants, athletic level, dependent variable, independent variable(s) associated with performance (correlation coefficient, *p-*value) or if the independent variables comprise a significant model (equation): the coefficient of determination (*R*^2^) and the standard error of the estimate (SEE), the limits of agreement of the Bland–Altman plot (only in half-marathon) and the predictive equation.

The tables present two types of study: those without a prediction equation in which they provide the correlations between the independent variables and the dependent variable (correlation coefficient and *p*-value. The studies including a prediction equation are shown in the tables with the *R*^2^ value and the SEE. In Table 4 only, corresponding to the studies on the half-marathon, a further section is included, pertaining to the information on bias between the predicted and the actual time, with the limits of agreement derived from the studies by Knechtle’s [8,18,19] and other authors [14,15,20,21]. Finally, the studies with a prediction equation are presented in a highlighted text box

### 3.8. Variables and Models Associated with the 5000 m Event

*Search*: The different keywords were combined as follows: “performance, performance prediction”, “performance determinants”, “anthropometric and physiological determinants”, “5000 m”, “5 km”.

*Appraisal*: The subjects of the different studies were generally moderately trained or highly trained athletes of different athletic levels (amateur, collegiate, competitive, elite), except for the study by Stratton which includes untrained individuals [22]. Of all the studies, only a few provide coefficients for determining the independent variable [13,23,24,25,26,27]. The coefficients of determination ranged from 0.62 to 0.98, but none of the studies reported the standard error. Additionally, the study by Stratton has an external validation study in a subsample of subjects [22].

*Synthesis*: It should be noted that in all the studies, the variables most used for performance prediction are derived from determinations of aerobic metabolism. In one study the variable is the percentage of fat mass measured by anthropometry [28] and in another the fat-free mass [29]. Only one study was conducted in which the velocity at VO_2_max in the University of Montreal Track Test, as a field variable, is presented as a predictor variable [13].

*Analysis*: Table 2 presents 12 studies from 1983 to 2015 [12,13,22,23,24,25,26,28,29,30,31,32]. The most notable are the physiological variables such as VO_2_max [12,23,25,32] and *v*VO_2_max, [13,22,28,31] and RE measurements [12,29,30,33].

Only one study examines training variables [26]. The most important anthropometric variables are the inclusion of body composition fractions (fat mass and fat-free mass). Of the 12 studies, eight include a prediction equation [12,22,23,24,25,26,28,29] (Table 2).

### 3.9. Variables and Models Associated with the 10,000 m Event

*Search*: The different keywords were combined as follows: “performance, performance prediction,” “anthropometric and physiological determinants,” “performance determinants,” “10,000 m,” “10 km”.

*Appraisal*: The subjects of the different studies were generally trained athletes of different levels (amateur, competitive, elite) with the exception of the studies by Brandon [34] and Berg [35], which included only moderately trained individuals.

*Synthesis*: In all the studies, the variables most used for prediction continue to be those derived from laboratory tests. Furthermore, these variables increase compared to the 5000 m specialty. New variables include those from training data, such as number of training sessions, miles per week and years of training [7]. In addition, anthropometric variables such as skinfolds [36] and two somatotype components are beginning to be included [35] although these equations have a low-moderate R^2^ (0.380–0.41).

*Analysis*:Table 3 presents 13 studies from 1983 to 2014 [13,23,26,27,28,33,34,35,36,37,38,39,40,41,42,43,44,45,46]. Physiological variables such as VO_2_max [23,32,33,34,38] and *v*VO_2_max continue to be prominent [27,28,33]. Of the 13 studies, seven have a prediction equation [7,23,26,28,34,37,44]. The coefficients of determination (R^2^) of the equations by Bale et al. (1986) are moderately high (from 0.75 to 0.86) and are based on training variables including the number of training sessions, miles run, years of training and a somatotype component such as ectomorphy [7,38] and the studies by Fay et al. (1989) with R^2^ > 0.84, based on the velocity associated with metabolic variables such as lactate at 2 and 4 mmol/L and at VO_2_max (Table 3).

### 3.10. Variables and Models Associated with the Half-Marathon Event

*Search*: The different keywords were combined as follows: “long distance runners,” “performance, performance prediction,” “anthropometric and physiological determinants,” “performance determinants,” “half-marathon”.

*Appraisal*: The subjects of the different studies were generally at an amateur level and infrequently at a competitive level (Roecker et al., 1998) [28].

*Synthesis*: It should be noted that the half-marathon is not an official specialty of the Olympic Games or the World Championships, although there are national and international competitions in this event. Consequently, the largest number of individuals who practice this modality are amateur runners, with different training loads, ages and levels of experience. Multiple associations have been found between performance and anthropometric variables, but with models of moderate predictive power (R^2^ = 0.440–0.71) and with wide limits of agreement between the predicted time and the actual race time. Finally, two studies should be mentioned due to the high coefficient of determination (R^2^ = 0.84) and relatively low limits of agreement obtained through the distance covered in the Cooper test as a predictor variable [14,15]. This is a simple field test that can be introduced into training routines and can provide very useful information and Cooper’s test has a good accuracy and reliability in amateur long-distance runners [20].

*Analysis*: Table 4 presents 11 studies from 1985 to 2020 [8,14,15,16,28,47,48,49,50]. Of these 11 studies, nine were undertaken from 2011. In this section we should note the many contributions by Knechtle’s group. Multiple publications by these authors base their results on the relationships between performance in half-marathon races with anthropometric variables such as skinfolds, estimated body composition variables such as fat mass and skeletal muscle mass, and training load variables such as average training velocity [8,48,50,51] (Table 4).

### 3.11. Variables and Models Associated with the Marathon Event

*Search*: The different keywords were combined as follows: “long distance runners,” “performance, performance prediction,” “anthropometric and physiological determinants,” “performance determinants” and “marathon”.

*Appraisal*: The subjects in the different studies are generally trained and/or highly trained and at different levels (amateur, competitive, elite), with the exception of the study by Hagan which includes novice runners [41].

*Synthesis*: The first studies in this field, by Foster (1983) [32], Slovic (1977) [52], Davies and Thompson (1979) [53], Föhrenbach et al. (1987) [39] and Noakes et al. (1990) [43], primarily relate training variables to athletic performance. A powerful prediction model should be mentioned (Tanda, 2011) [54], which estimates race pace with a high coefficient of determination of 0.81.

*Analysis*: Table 5 presents 21 studies from 1975 to 2020. Of note are the variables associated with exercise physiology and aerobic metabolism [28,40,41,53] as well as, to a large extent, those related to training load [26,41,52,54,55,56] (Table 5).

## 4. Discussion

The main strength of this literature review is the considerable number of publications and the subsequent analysis of the variables that make up the prediction equations of each of the specialties. This analytical text invites the reader and the scholar to use the assessment methods available to evaluate athletic performance.

One of the difficulties we encountered in comparing the different equations is that there is no consensus on the definition of the type of athletes, with each author having named the type of subjects involved. Therefore, we recommend unifying and clearly defining each of the athletes and their level. We also found great differences in the number of athletes participating in the studies, ranging from eight subjects [24,36] to 427 including both men and women [28].

The dependent variables of the models found are diverse, as they are expressed as time in minutes, seconds, hours; speed in m/s, m/min, km/h and, finally, the race pace in s/km. On this issue these have been the independent variables that have defined training loads, without finding work that has influenced in a quantification of both, strength trainings [57] and high-intensity intervals [6,58] from which predictor variables can be extracted. The number of independent variables is two or three, with some equations having as many as six independent variables. A piece of data missing in almost all the studies is the variance inflation factor (VIF), which informs us of multicollinearity.

Some of the possible solutions to the problem of multicollinearity are the following: improvement in the sample design by extracting the maximum information from the observed variables, elimination of the variables suspected of causing multi-collinearity and, finally, in the case of having few observations, increasing the sample size [59].

The identification of physiological variables for performance prediction has at least two important applications around sports training. The first is the evaluation of certain defining physiological characteristics related to the sports specialty and the second is associated with training (volume and intensity) in relation to the sports modality and especially with regard to metabolic and functional characteristics (capacity and power, aerobic and anaerobic).

The most widely studied variables for predicting aerobic performance in running are VO_2_max and *v*VO_2_max, both of which are fundamentally associated with short distances such as the 5000 m and 10,000 m events [10,22,23,25,28,43]. This is likely because the intensities at which these races are executed are very close to maximal intensities and thus their close correlation. VO_2_max is the physiological variable that represents aerobic capacity, or in other words, the measurement of the maximum energy produced by aerobic metabolism per unit of time. Both *v*VO_2_max and VO_2_max would effectively be the same as they occur essentially at the same time [28,31,43,60,61].

The variables related to the submaximum level and the variable intensities that occur in these areas have been studied extensively in all specialties, except for the half-marathon [26,28,39,43,62]. This is related to the fact that the half-marathon has not been recognized in the international federative sphere and, therefore there has been no interest in its study. In the half-marathon specialty, very few studies are available: one by Campbell in 1985 [47] and another by Roecker et al. [28] Campbell finds moderate-low correlations between some basic anthropometric parameters and running pulse rate and weeks of training. Roecker et al. [28] observed high correlations (r > 0.89) between individual anaerobic threshold and running velocity at an intensity of 4 mmol/L, both physiologically very similar concepts, and *v*VO_2_max. From 2011 onwards, the following references are provided by Knechtle’s group, which published many articles linking half-marathon times with numerous anthropometric variables and with low-moderate correlation coefficients [48] and with prediction models also with moderate coefficients of determination [19].

Many studies in the literature analyse performance prediction in aerobic specialties based on the physiological parameters mentioned above. However, these studies, using simple or multiple regression models, analyse the associations between physiological parameters and aerobic performance capacity in athletes for a single distance (frequently between 1500 m and 10,000 m) [27,61,63]

Based on the studies mentioned above, it has been proposed that race distance and, therefore, exercise intensity may influence the associations between physiological indicators and aerobic performance. Nonetheless, no studies have addressed aerobic performance capacity in the same athletes at different distances with two or more physiological indicators, particularly in studies with *v*VO_2_max and its respective time to exhaustion. As a result, it is not possible to draw the same conclusions for all sports specialties and at different athletic levels (amateur, highly trained, trained) [60]. The variables related to the quantity and quality of training are almost exclusive to studies undertaken in the marathon specialty and for different levels of training.

A contribution of this review is the general idea that the parameters recorded at the end of the graded exercise stress test are well understood, as are the parameters associated with aerobic and anaerobic thresholds, in terms of both metabolism and gas exchange, since in the different prediction models, variables range between 85% and 99% of the stress intensities. From our point of view, it is here, in this range of intensities where stronger associations should be sought, that would allow us to obtain more powerful models for predicting performance.

Similarly, in the field of ultramarathon races, which are becoming increasingly popular, variables related to RE, associated low lactate concentrations, percentage of VO_2_max and the search for models that integrate genetic aspects related to muscle damage and protein synthesis capacity should be explored, as well as how to more accurately determine and calculate training load both in terms of quantity and quality. In relation to genetic studies, it has been shown that polymorphisms (about 160) in 27 genes were identified in 10,442 participants, of whom 2984 were marathon runners, leaving the variance in the result on sports performance to be studied [64].

### 4.1. Practical Applications

The prediction of race time in the long-distance modalities has, above all, an initial application for novice runners, who have little knowledge of their race paces, allowing them to adjust to constant paces. Running paces can be modified depending on the phase of training. The knowledge of the variables associated with performance in long-distance runners should help coaches and exercise physiologists understand and promote the search for new variables that improve the prediction of sports performance.

### 4.2. Future Research Directions

**Figure 2:**
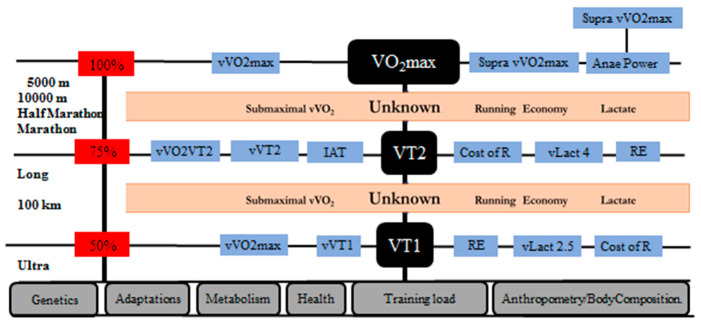
Proposal for the study of long-distance runners.

As future lines of research, we must consider aspects that are currently known as physiological events that occur at the aerobic threshold (VT1), at the anaerobic threshold (VT2) and at maximum intensities (VO_2_max). At the lactate threshold, normally below 50–60% of VO_2_max, we know the lactate values, the energy expenditure for the race and the RE. These same parameters are also well known at the anaerobic threshold, which could be estimated to be around 85% of VO_2_max. We have many parameters that associate sports performance with VO_2_max, such as running speed, individual anaerobic threshold, and lactate levels. In addition, we know the physiological responses when reaching 100% of VO_2_max. Up to this point we can see what the exercise physiology studies have been based on for performance. However, we believe that there is a gap in what occurs between the aforementioned points, with regard to studying these values (percentage VO_2_max, RE, lactate levels, etc.). Anaerobic capacities should also be further explored, particularly as related to the 5000 and 10,000 m events. Finally, we must not forget the quantification of training load and of the molecular and genetic aspects related to human performance (see Figure 2).

## 5. Conclusions

Physiological stress assessments are almost exclusive to the short long-distance specialties (5000 m and 10,000 m). Half-marathon predictor variables are mainly anthropometric, with moderate coefficients of determination and physiological and field test variables with high coefficients *R*^2^. The most relevant variables in the marathon modality are training variables derived from the evaluation of aerobic metabolism and anthropometric parameters.

## Figures and Tables

**Table 1:** Partial and total figures for performance prediction variables in long-distance specialties.

	Long-Distance Specialties					
Variables	5000 m	10,000 m	HM	M	Total	% of Total
Demographic	4	1	1	1	7	*5.1*
Aerobic Metabolism	26	14	3	16	59	*43.4*
Training	1	5	2	28	36	*26.5*
Anthropometry	2	5	16	5	28	*20.6*
Field test	0	1	2	0	3	*1.47*
Others	0	1	0	3	4	*2.94*
Subtotals/*Total*	33	27	24	51	137	*100*

HM: Half-marathon, M: Marathon.

**Table 2:** Multiple regression models associated with performance in 5000 m races.

Author	Year	Sex	*n*	Level	Dependent Variable	Independent Variable	r	*p*	R^2^	SEE
Foster	1983	1	28	Well-trained	3 Miles	VO_2_max	−0.92			
						Training volume				
						Intensity				
Tanaka	1984	1	21	Trained	5000 m	*v*VO_2_max	0.78	<0.001	0.62	nr
Ramsbottom	1987	1	55	University	VO_2_max	5000 m	−0.85	<0.01		
		0	43			5000 m	−0.80	<0.01		
	1987	1	55	University	5000 m	RE	0.39	<0.01		
		0	43			RE	0.34	<0.05		
Fay	1989	0	13	Mod-Highly	5000 m (m/min)	Vlact 4 mMol/L (m/min)	0.94		0.940–0.97	nr
						VO_2_max (ml/kg/min)				
						Oxygen cost of running	−0.4–(−0.63)			
	**Velocity (m/min) = 0.346 (vLac 4 mMol/L) + 1.899 (VO_2_max)**									
Kenney	1985	1	8	Elite	5000 (time in sec)	Age + VT2 (mL/kg/min)		<0.02	0.98	nr
	**Time (sec) = 11555 − 5.1 (age) − 2.9 (VT2)**									
Weyand	1994	1–0	22–19	Competitive	5000 m	Peak O_2_ Def (POD)	−0.4			
						VO_2_max	High			
						%VO_2_ AT				
						RE at 3.6 m/s				
						Gender (1 = male; 2 = female)				
						Specialty				
	**Time (sec) = 0.38 (POD) − 1.29 (VO_2_max) + 1.25 AT(%VO_2_)**									
	**+ 4.42 (RE) + 55.9 (Gender) − 47.4 (specialty)**									
	**(1 sprinter, 2 long-distance runner) + 1664.9**								nr	nr
Takeshima	1995	1	51	Popular	5000 m (m/s)	VO_2_ LT (ml/kg/min)			0.87	
						Age				
						ARD			0.89	
						VO_2_ LT (ml/kg/min)			0.79	
						Age				
						VO_2_ LT (ml/kg/min)			0.82	
						Age				
						ARD				
	**Velocity (m/s) = 4.436 + 0.045 (VO_2_ LT) − 0.033 (Age) + 0.005 (ARD)**						0.89			0.27
Roecker	1998	1–0	339–88	Competitive	5000 m (m/s)	vPeak (km/h)	0.91	<0.001	0.940–0.97	
						IAT (m/s)	0.91			
						% Fat Mass				nr
						MHR (bpm)				
						Max Lact (mMol/L)				
	**Velocity (m/s) = 3.404 + 0.683 (vPeak) + 0.274 (IAT) − 0.05 (%FM)**									
	**(MHR) − 0.079 (Max Lact)**									
Nummela	2006	1	18	Well-trained	Velocity (m/s)	VO_2_max	0.55	<0.05		
						MART				
	**Vel (m/s) = 0.066 (VO_2_max) + 0.048 (MART) − 0.549**								0.728	nr
Stratton	2009	1–0	17–22	Untrained	5000 m (km/h)	VO_2_ max (ml/kg/min)	0.55	<0.01		
						V LT (km/h)	0.73	<0.01		
						V Max (km/h)	0.89	<0.01		
	**Run velocity (km/h) = −1.124 + 0.514 (Vmax) + 0.267 (V LT)**								0.812	
	2009	1–0	17–22	Trained	5000 m (km/h)	VO_2_ max (ml/kg/min)	0.51	<0.01		
						V LT (km/h)	0.76	<0.01		
						V Max (km/h)	0.83	<0.01		
	**Run velocity (km/h) = −2.629 + 0.546 (Vmax) + 0.345 (V LT)**								0.738	
Mendes de Souza	2014	1	10		5000 m	*v*VO_2_ max Lab		0.05	0.35	nr
		1	10		5000 m	*v*VO_2_ max Montreal		0.002	0.66	nr
Dellagrana	2015	1	23	Moderately trained	5000 (time)	vVT (km/h)	−0.64	0.001		
						RE at 11.2 km/h (L/min)	0.44	0.035		
						Fat-free mass (kg)	0.57	<0.005		
	**5 km T (min) = 25.64 − 0.71 (vVT) − 3.38 (RE 11.2) + 0.21 (FFM)**								0.71	0.67

r: correlation coefficient; *p*: significance level; R^2^: coefficient of determination; SEE: standard error of estimation; *v*VO_2_max: max velocity in VO_2_max; RE: running economy; VLact4: velocity at 4mMol/L; AT: anaerobic threshold; POD: peak oxygen deficit; LT: lactate threshold; ARD: average running duration; IAT: individual anaerobic; threshold; MHR: maximal heart rate; Max Lact: maximal lactate; MART: maximal anaerobic running test; *v*VO_2_maxLab: maximal velocity at exercise laboratory test: *v*VO_2_max Montreal: maximal velocity on Montreal field test. vVT: velocity at ventilatory threshold.

**Table 3:** Multiple regression models associated with performance in 10,000 m races.

Author	Year	Sex	*n*	Level	Dependent Variable	Independent Variable	r	*p*	R^2^	SEE
Foster	1983	1	17	Well-trained	3 Miles	VO_2_ max	−0.94			
						Training volume				
						Intensity				
Tanaka	1984	1	21	Trained	10,000 m	*v*VO_2_ max			0.96	nr
		1	21	Trained	10,000 m	vAT (ml/kg/min)	0.80	<0.001		
Bale	1986	1	60	Elite & Good	Time 10,000 m	Workouts (WO)per week	−0.87		0.75	2.28
	**Time (min) = 44.27 − 1.44 (WO)**									
						WO + Miles (MW) per week	−0.84			
	**Time (min) = 46.32 − 0.91 (WO) − 0.11 (MW)**								0.8	2.08
						WO + MW + Running years (RY)	−0.80			
	**Time (min) = 46.45 −0.68 (WO) − 0.11 (MW) − 0.38 (RY)**								0.83	1.92
						WO + MW + RY + Ectomorphy	−0.40			
	**Time (min) = 47.93 − 0.68 (WO) − 0.10 (MW) – 0.38 (RY) − 0.68 (Ectomorphy)**								0.86	1.78
Brandon	1987			Middle	10,000 (m/s)	VO_2_max (ml/kg/min)				
						Anaerobic Capacity (AC)				
						Height (cm)				
	**10,000 (m/s) = 127.39 + 0.64 (VO_2_) + 0.21 (AC) + 0.4 (Height)**									
Fay	1989	0	13	Moderate	10,000 m (m/min)	Vlact 4 mmol/L(m/min)			0.840–0.94	
				High		VO_2_max (ml/kg/min)				
						Vlact 2 mmol/L(m/min)				
	**10,000 (m/min) = 0.437 (vLA 4 mmol/L) + 2.082 (VO_2_max) + 8.698**									
	**10000 (m/min) = 0.728 (vLac 4 mmol/L) + 57.926**									
	**10,000 (m/min) = 0.407 (vLac 2 mmol/L) + 2.276 (VO_2_max) + 12.706**									
Morgan	1989	1	10	Well-trained	Time (min)	VO_2_max	−0.45	>0.05		
						*v*VO_2_max	−0.87	<0.01		
						Vel at 4 mmol/L	−0.82	<0.01		
						RE	0.64	<0.05		
Petit	1997	1	15	Trained		Vel Ventilatory threshold	0.95		0.96	
						Vel HR def (km/h)				
	**10,000 (km/h) = 1.03 (Vel Deflection HR)**									
Berg	1998	1	34	Mod trained	Time 10,000 m	BMI and Mesomorphy	0.61		0.38	7.3
	**10,000 (min) = 4.12 (BMI) − 4.5 (Mesomorphy) − 29.1**									
		0	19	Mod trained	Time 10,000 m	Endomorphy	0.64		0.41	6.5
	**10,000 (min) = 37 + 3.3 (Endomorphy)**									
Evans	1995	0	31	Highly trained	10,000 Pace (m/min)	VO_2_max	0.89	0.05	0.8	
						Lac Threshold	0.89	0.05	0.8	
						VO_2_ (ml/kg FFM/min)	0.81	0.05	0.66	
						VO_2_ in LT	0.84	0.05	0.71	
Takeshima	1995	1	51	Trained	10,000 vel (m/s)	VO_2_ in LT (ml/kg/min)	0.78		0.62	nr
						Age				
						VO_2_ in LT	0.81		0.67	
						Age				nr
						Workout (min)				
	**10,000 (m/s) = 4.371 + 0.037 (VO_2_ in LT) − 0.031 (Age) + 0.005 (Workout)**						0.82			0.335

r: correlation coefficient; *p*: significance level; R^2^: coefficient of determination; SEE: standard error of the estimate; VO_2_max: maximal oxygen uptake; *v*VO_2_max: velocity at VO_2_max; WO: workouts; vAT: velocity at anaerobic threshold; Lact 4: velocity at 4 mmol/L; vLact 2: velocity at 2 mmol/L; RE: running economy; vHR def: velocity at heart rate deflection; BMI: body mass index; FFM: fat-free mass; LT: lactate threshold; AT: anaerobic threshold; IAT: individual anaerobic threshold; HR: heart rate; Max Lact: maximal lactate; SK: skinfold.

**Table 4:** Multiple regression models associated with performance in half-marathon races.

Author	Year	Sex	*n*	Level	Dependent Variable	Independent Variable	r	*p*	*R* ^2^	SEE	L LOA	to	U LOA
Campbell	1985	1–0	88–10	Finishers	Running Speed (km/h)	Age							
						Height	0.18	ns					
						Pulse rate 1 (PR1)	−0.53						
						Pulse rate 2 (PR2)	−0.35						
						km/week (K)	0.53	<0.01					
						Training weeks (NW)	0.4	<0.01					
						BMI	−0.41	<0.01					
	**Running Speed (km/h) = 21.3 +0.028 (K) − 0.31 (BMI) − 0.037 (PR2) + 0.012 (NW)**								0.47	nr			
Roecker	1998	1–0	339–88	Competitive		IAT	0.93	<0.001					
						Running vel at 4 mmol/L	0.91	<0.001					
						*v*VO_2_max	0.89	<0.001					
Rüst	2011	1	84	Recreational	Race time	BMI	0.56	0.01					
						Skinfolds	0.360–0.53	0.005					
						Percent fat mass	0.49	0.01					
	**Race time = 72.91 + 3.045 (BMI) − 3.884 (SRT)**								0.44	nr	−25.1	to	25.1
Knechtle	2011	0	42	Recreational	Race time	Skinfolds	0.490–0.61	<0.001					
	**Race time = 166.7 + 1.7 (mid axilla SK) − 6.4 (SRT)**								0.71	nr	nr		nr
Muñoz	2013	1	24		Vel (km/h)	Velocity 2 at 14.6 ± 2.6 km/h							
						Blood Lactate at velocity 2			0.97	0.414			
	Vel Half-marathon (km/h) = V2 * 1.085 + (BLa2 * −0.282) − 0.131									nr			
Friedrich	2014	0	83	Recreational	Race time	Weight	0.63	<0.0001					
						Height	0.27	0.01					
						BMI	0.57	<0.0001					
						Circumferences	0.510–0.55	<0.0001					
						Skinfolds	0.390–0.59	<0.0001					
						Skeletal Muscle mass	0.24	0.03					
						Fat mass	0.6	<0.0001					
Friedrich	2014	1	147	Popular	Race time	Weight	0.27	0.0009					
						Height	−0.17	0.04					
						BMI	0.46	<0.0001					
						Arm circumference	0.37	<0.0001					
						Skinfolds	0.290–0.43	<0.0001					
						Skeletal Muscle mass	−0.07	>0.05					
						Fat mass	0.49	<0.0001					
Knechtle	2014	1	147	Recreational	Race time (min)	Percent fat mass							
						SRT (km/h)							
	**Race time (min) = 142.7 + 1.158 (%FM) − 5.223 (SRT)**								0.42	13.3	−26	to	25.8
Knechtle	2014	0	83	Recreational	Race time (min)	Percent fat mass							
						SRT (km/h)							
	**Race time (min) = 168.7 + 1.077 (%FM) − 7.556 (SRT)**								0.68	9.8	−19	to	19.1
Gómez	2017	1	48	Recreational	Race time (min)	Week training (km) WT	−0.75	< 0.05					
						Running experience (years) RE	−0.80	< 0.05					
						BMI	0.64	< 0.05					
						Sum 6 Skinfolds (mm)	0.78	< 0.05					
	**Race time (min) = 56.83 − 0.11 WT − 0.46 RE + 1.19 BMI + 0.16 Sum6SKF**								0.82	nr	−9.2	to	12.2
	2017	1	48	Recreational	Race time (min)	Peak speed (km/h)	−0.92	< 0.05					
						RCT (km/h)	−0.92	< 0.05					
	**Race time (min) = 180.86 − 2.81 Peak speed − 2.77 RCT**								0.90	nr	−6.7	to	6.4
	2017	1	48	Recreational	Race time (min)	RCT Step rate (Hz)	−0.38	< 0.05					
						RCT Step length (m)	−0.87	< 0.05					
						Maximal step length (m)	−0.73	< 0.05					
	**Race time (min) = 271.9 − 33.38 RCTsr − 28.38 RCTsl − 29.8 Msl**								0.88	nr	−9.7	to	5.7
	2017	1	48	Recreational	Race time (min)	Peak speed (km/h)	−0.92	< 0.05					
						RCT (km/h)	−0.92	< 0.05					
						Running Experience (years)	−0.75	< 0.05					
	**Race time (min) = 169.54 − 2.51 Peak speed − 2.25 RCT − 0.37 RE**								0.93	nr	−6.7	to	6.0
Alvero-Cruz	2019	1	23	Recreational	Race time (min)	Cooper test (m)	−0.92	<0.0001					
	**Race time (min) = 201.26 − 0.03433 Cooper (m)**								0.873	3.78	−7.5	to	7.4
	2019	1	23	Recreational	Race time (min)	*v*VO_2_max (km/h)	−0.85	< 0.0001					
						Weight (kg)	0.4	0.04					
	**Race time (min) = 156.7117 − 4.7194 *v*VO_2_max − 0.3435 Weight**								0.769	5.28	9.5	to	9.7
Alvero-Cruz	2020	1	177	Recreational	Race time (min)	Cooper test (m)	−0.906	<0.0001					
		0	21	Recreational									
	**Race time (min) = 205.6272 − 0.0356 Cooper (m)**								0.82	5.19	−10.7	to	9.7

r: correlation coefficient; *p*: significance level; *R*^2^: coefficient of determination; SEE: standard error of the estimate; L: Low; U: Upper, LOA: limits of agreement; nr: no reported; BMI: body mass index; IAT: individual anaerobic threshold; *v*VO_2_max: velocity at VO_2_max; SRT: speed running time.

**Table 5:** Multiple regression models associated with performance in marathon races.

Author	Year	Sex (M/F)	*n*	Level	Dependent Variable	Independent Variable	r	*p*	R^2^	SEE
Foster	1975				Race Time (min)	VO_2_max(ml/kg/min)				
	**Time (min) = 3.45 (VO_2_max) + 387.3**						nr		nr	
Foster	1975				Race Time (min)	VO_2_max				
						Training longer in last 8 w				
						Pace (seconds/mile)				
	**Time (min) = 2.75 (VO_2_max) − 0.022 (miles 8w) − 1 (TL8w) + 0.146 (pace) + 319.4**						nr		nr	
Slovic	1977				Race Time (min)	Best record in mile (min) (BR1)				
						Best record in 5 miles (min) (BR5)				
						Best record in 10 miles (min)(BR10)				
						Miles in last 8 weeks				
						Finisher of one marathon				
						Training longer in last 8 w				
	**Time (min) = 0.45 (BR1min) − 7.9 (Finisher) − 0.08(Miles 8w) − 1.45 (TL8w(min) + 116.5**						nr		nr	
Slovic	1977				Race Time (min)	Best record in 5 miles (min) (BR5)				
						Miles in last 8 weeks				
						Training longer in last 8 w				
	**Time (min) = 6.62 (BR 5min) − 0.05(Miles 8w) − 1.45 (TL8w(min)) + 42.8**						nr		nr	
Slovic	1977				Race Time (min)	Best record in 10 miles (min)(BR10)				
						Miles in last 8 weeks				
						Training longer in last 8 w				
	**Time (min) = 2.98 (BR 10 (min) − 0.04(Miles 8w) − 1.3 (TL8w(min) + 46.6**						nr		nr	
Davis	1979				Race Time (min)	VO_2_max(ml/kg/min)				
						%VO_2_ in AT				
	**Time (h) = 7.445 − 0.0338 (VO_2_max) − 0.0303 (%VO_2_)**						0.99			
Hagan	1981	1	50	Trained	Race Time (min)	VO_2_max	−0.63			
						Avg km WO in last 9 weeks	−0.64			
						total km	−0.67			
						overall WO in last 9 weeks	−0.62			
						Mean pace (m/min)				
	**Time (min) = 525.9 + 7.09 km (kmWO) − 0.45 (WO speed m/min) − 0.17 (km 9 weeks)**								0.71	
	**−2.01 (VO_2_max, ml x kg^−1^ x min^−1^) − 1.24 (age, year)**									
Foster	1983	1	25	Well-trained	26.2 miles	VO_2_max	−0.95			
						Training volume				
						Intensity				
Bale	1985	0	36	Trained	Race Time (min)	workouts/week				
	**Time (min) = −4.42 (WO per week) + 218.5**						nr		nr	
	1985	0	36	Trained	Race Time (min)	workouts/week				
						Ectomorphy				
	**Time (min) = −3.72 (WO per week) − 7.02 (Ectomorphy) + 242.6**						nr		nr	
	1985	0	36	Trained	Race Time (min)	workouts/week				
						Ectomorphy				
						training years (TY)				
	**Time (min) = −3.32 (WO per week) − 6.05 (Ectomorphy) − 0.85 (TY) + 240.6**						nr		nr	
Hagan	1987	0	35	Combined	Race Time (min)	Mean km/day	0.77	<0.001	0.59	
						Training pace (m/min)	0.66	<0.001	0.44	
	**Race Time = 449.88 − 7.61 (Mean km/day) − 0.63 (Training pace m/min)**						0.82	nr	0.68	18.4
		0	16	Experienced	Race Time (min)	BMI	0.7	nr	0.49	
						Training pace (m/min)	0.78	<0.001	0.61	
	**Race Time = 214.24 + 393.07 (BMI) − 0.68 (training pace m/min)**						0.87	nr	0.76	12.4
		0	19	Novice	Race Time (min)	BMI	0.31	ns	0.1	
	**Race Time = 369.58 − 10.1 (Mean km/day)**						0..69	nr	0.48	22.2
Föhrenbach	1987	1–0	34		Race Time (min)	Mean km last 9 weeks				
						vLact 2,5 (m/s)	0.880–0.99	<0.001		
						vLact 3 (m/s)				
						vLact 4 (m/s)				
Noakes	1990	1	20		Race Time (min)	Time in Half-M (THM)				
						Lact AT (mmol/L)				
						% peak Vel in AT (lact)	−0.88			
	**Time (min = 1.98 (THM) + 6.23 AT (mmol/L) − 0.46 AT % vPeak mmol/L + 33.84**									
	**Time (min) = 1.94 (THM) + 5.8 AT (mmol/L) − 0.44 AT % vPeak mmol/L + 0.39 RE at 16 km/h + 16.79**									
	**Time (min) = 1.29 % vPeak mmol/L − 10.86 vLT (km/h) + 241.3**									
	**Time (min) = −4.92 vLT (km/h) − 4.46 vPeak (km/h) + 337.8**									
Noakes	1990	1	20		Race Time (min)	Time in Half-M				
						Lact AnT (mmol/L)				
						% peak Vel i nAT (lact)				
						VO_2_ at 16 km/h	0.760–0.9			
					Race Time (min)	Lact AnT (mmol/L)				
						% peak Vel in AT (lact)				
					Race Time (min)	Vel in AnT by lact in km/h				
						*v*VO_2_max (km/h)				
Takeshima	1995	1	51	Popular	Mean Velocity (m/s)	VO_2_ LT (ml/kg/min)				
						Age				
						Mean Duration Workouts (min)				
	**Mean Vel (m/s) = 0.038 (VO_2_ LT) − 0.031 (Age) + 0.005 (MDWO) + 3.707**						0.93			0.199
Roecker	1998	1–0	339–88	Competitive	Mean Velocity (m/s)	vIAT (m/s)	0.93	<0.001	0.950–0.97	
						*v*VO_2_max (km/h)	0.87	<0.001		
						MHR				
						Weight				
	**Mean Vel (m/s) = 0.546 (vIAT) + 0.293 (*v*VO_2_max) + 0.013 (km/week) − 0.0155 (MHR) − 0.0253 (Weight) + 3.4**									
Arrese	2006	0	8	Highly trained	Race Time	Iliac crest SK	0.76	<0.05		
						Abdominal SK	0.76	<0.05		
						Subscapular SK	0.78	<0.05		
						Serum ferritin (µg/L)	−0.76	<0.05		
	**Race Time = 7658.331 + 55.519 (Subscapular SK) − 4.834 (ferritin) + 34.895 (Sum 6 SK)**								0.992	<0.001
	2006	1	10	Highly trained	Race Time	Left ventricular diameter (LVD)	−0.68	<0.05		
						Lactate at 10 km/h	0.91	<0.001		
						Lactate at 22 km/h				
	**Race Time = 8408.623 (lact 10 km/h) − 18.255 (LVD) + 22.522 (lact 22 km/h)**								0.991	<0.001
Tanda	2011	1–0	21–ene	Trained	Pace (sec/km)	K (km/week)	0.94		0.81	
						Pace (P) (sec/km)			0.85	
	**Pace (sec/km) = 17.1 + 140 exp [–0.0053 *K*] + 0.55 (Pace)**								0.81	5.77
Muñoz	2013	1	24		Vel (km/h)	Velocity 1 at 13,5 ± 0,9 km/h (V1)				
						Blood Lactate at velocity 1			0.81	0.626
	**Vel Marathon (km/h) = V1 1.085 + (BLa2 − 0.429) − 0.170**									
Tanda	2013	1	126	Recreational	Pace (sec/km)	Km week				
						Pace training (sec/km)				
						Percent body fat				
	**Pace (sec/km) = 11.03 + 98.46 exp [−0.0053 Km week] + 0.387 (Pace) + 0.1 exp [0.23 %BF]**						0.81		0.64	14.3
Mooses	2013	1	20	International	IAAF scoring	Total time on treadmill (TtT)(sec)			0.40	66.2
	**IAFF score = 162.30 + 0.41 (TtT)**									
Till	2016	1–0	40	Recreational	Race Time (min)	treadmill time (min)				
	**Time (min) = −3.85 (treadmill time) +351.57**								0.447	
Salinero	2017	1	84	Amateur	Time (min)	% Body fat (%BF)	0.42	<0.001		
						∆ Recovery Ruffier test (RT)	0.37	<0.000		
						Half-marathon performance (HMP)	0.81	<0.001		
	**Time (min) = 96.1 + 2.3 (%BF) + 62.9 (RT) + 0.023 (HMP)**								0.59	nr
					Time (min)	% Body fat (%BF)	0.42	<0.001		
						∆ Recovery Ruffier test (RT)	0.37	<0.000		
						10 km performance (10 km P)	0.73	<0.001		
	**Time (min) = 104.3 + 3.1 (%BF) + 67.3 (RT) + 0.045 (10 km P)**								0.53	nr
Esteve-Lanao	2019	1–0	8–8	Recreational	Avg speed 42k (km/h)	116 days before = AnT	0.810–0.94	<0.05		
	**Speed 42k (km/h) = SpeedAnT (km/h) 0.771 + 0.959**								0.659	nr
						88 days before = AnT				
	**Speed 42k (km/h) = SpeedAnT (km/h) 0.863 − 1.463**								0.714	nr
						60 days before = AnT				
	**Speed 42k (km/h) = SpeedAnT (km/h) 1.013 − 0.944**								0.76	nr
						32 days before = AeT				
	**Speed 42k (km/h) = SpeedAeT (km/h) 1.012 − 1.147**								0.804	nr
						11 days before = AeT				
	**Speed 42k (km/h) = SpeedAeT (km/h) 1.004 − 1.145**								0.85	nr
Keogh	2020	1–0	157–103	Recreational	Time (min)	Age				
						BMI				
						Marathon experience (ME)				
						Predicted finish time (PFT)				
						Diff pred vs. finish time (DPvF)				
						Pace St deviation				
						Sex				
	**Time (min) = −5.252 + 0.162 Age + 0.319 BMI + 0.451 ME + 0.947 PFT − 0.636 (DPvF) + 2.925 Pace − 3.232 Sex**								0.858	nr

r: correlation coefficient; *p*: significance level; *R*^2^: coefficient of determination; SEE: standard error of estimation; VO_2_max: Maximal oxygen uptake; %VO_2_AT: percentage of VO_2_max at anaer. threshold; Avg km WO: average km of workouts; BMI: body mass index; vLact 2.5: velocity in m/s at 2.5 mmol/L; vLact 3: velocity in m/s at 3 mmol/L; vLact 4: velocity in m/s at 4 mmol/L; AnT: anaerobic threshold; MHR: maximal heart rate; *v*VO_2_max: velocity at VO_2_max; LVD: left ventricular diameter.
